# Antimelanogenic and Antioxidant Effects of Postbioics of *Lactobacillus* Strains in &alpha;-MSH-Induced B16F10 Melanoma Cells via CREB/MITF and MAPKs Signaling Pathway

**DOI:** 10.4014/jmb.2408.08015

**Published:** 2024-09-30

**Authors:** Hye-Won Lee, Yu-Rim Lee, Kyung-Min Park, Na-Kyoung Lee, Hyun-Dong Paik

**Affiliations:** Department of Food Science and Biotechnology of Animal Resources, Konkuk University, Seoul 05029, Republic of Korea

**Keywords:** *Lactobacillus*, antimelanogenic, antioxidant, CREB/MITF signaling pathway, MAPK signaling pathway

## Abstract

Abnormal melanin synthesis can lead to severe skin problems. This study investigated the anti-melanogenic effects on α-melanocyte stimulating hormone (α-MSH)-induced B16F10 cells using cell-free supernatants of *Lactiplantibacillus plantarum* WB326 and *Levilactobacillus brevis* WB2810. Samples were prepared using 1 mg/ml freeze-dried culture supernatant. Cell viability was assessed using B16F10 cells and MTT assay. Tyrosinase inhibition and melanin content decreased in the samples compared to those treated with α-MSH. This effect was also observed when L-DOPA staining was used under a microscope. Moreover, the mRNA expression levels of microphthalmia-associated transcription factor (MITF), tyrosinase, tyrosinase-related protein (TRP)-1, and TRP-2 decreased in the sample-treated group. Protein expression of the CREB/MITF/MAPK signaling pathway was also reduced. Using HPLC analysis, lactic and acetic acids were detected in the culture supernatants. Finally, the antioxidant effects of the samples were confirmed by comparison with those of Trolox and arbutin. According to the experimental results, their utilization is possible in the fields of functional materials and cosmetic ingredients.

## Introduction

Postbiotics, including bacterial lysates and ferments, are products derived from non-living microorganisms or their components offering health benefits independent of live bacteria [1]. Postbiotics are becoming increasingly popular in the cosmetics industry as ingredients or components of products [2]. According to Dou *et al*. [3], there has been an increase in the use of probiotics in skin-lightening cosmetics, leveraging components, such as lactic acid, found in lactic acid bacteria (LAB), which directly inhibit melanin formation by targeting tyrosinase (TYR). Owing to their minimal toxicity and efficient absorption, tyrosinase inhibitors produced by probiotics are considered promising candidates for skin-whitening agents [3]. Lactic acid produced by *Lactobacillus* can directly suppress melanin synthesis by downregulating tyrosinase activity and melanin synthesis by affecting tyrosinase expression or tyrosinase-related protein (TRP)-1 and TRP-2 to exhibit a brightening effect on the skin [4].

Melanogenesis is a crucial metabolic process in living organisms that relies on the enzymatic activity of TYR in melanocytes [5]. TYR is a copper-containing enzyme that catalyzes the conversion of tyrosine to levodopa, an intermediate of melanin biosynthesis [6]. This enzyme is pivotal in regulating the rate of melanin production and is the primary target for inhibiting melanogenesis [6]. The concentration, quantity, and distribution of melanin profoundly influence the color of the skin, eyes, hair, and animal shells [5]. Melanin not only serves as a pigment determinant but also plays a vital role in shielding against UV-induced skin damage [7]. Chronic exposure to UV radiation can disrupt melanin production, resulting in hyperpigmentation disorders such as freckles, melasma, and age spots [8]. This intricate process occurs within melanosomes during melanin formation [9].

α-MSH triggers the activation of the cAMP response element-binding protein (CREB) pathway, which in turn controls the microphthalmia-associated transcription factor (MITF) [34]. MITF is subject to regulation by multiple signaling pathways, including the cyclic adenine monophosphate (cAMP), mitogen-activated protein kinase (MAPK), and phosphoinositide 3-kinase/protein kinase B (PI3K/AKT) pathways [28]. The phosphorylation of members of the mitogen-activated protein kinase (MAPK) family, including p38 MAPK, ERK, and JNK, plays a crucial role in regulating MITF [30].

Natural melanin inhibitors have been attracting increasing attention as skin-whitening agents are surpassing the interest in chemically synthesized compounds such as arbutin, kojic acid, and nicotinamide [10]. However, effective synthetic inhibitors should be used with caution because of their potential cytotoxicity and carcinogenic effects [11]. Goelzer Neto *et al*. [12] actively researched various natural whitening agents, such as *Morus alba* and *Euphorbia supina*.

*Lactiplantibacillus plantarum* WB326 and *Levilactobacillus brevis* WB2810 were isolated from traditional Korean foods. This study aimed to investigate the potential development of cosmetic materials with skin-whitening and antioxidant functions using *L. plantarum* WB326 and *L. brevis* WB2810 by conducting antioxidant assays and assessing their ability to inhibit melanin production in melanoma cells.

## Materials and Methods

### Bacterial Strains and Culture Condition

*L. plantarum* WB326 and *L. brevis* WB2810 were isolated from kimchi. Arbutin was purchased from Daejung (Republic of Korea). *L. plantarum* WB326 and *L. brevis* WB2810 were cultured in de Man, Rogosa, and Sharpe (MRS; Difco Laboratories, USA) broth at 37°C for 24 h. After incubation, the bacterial cells were centrifuged at 14,240 ×*g* at 4°C for 5 min. The supernatant was collected and freeze-dried to obtain the probiotic sample.

### Cell Culture Condition

B16F10 melanoma cells (KCLB 80008) were obtained from the Korean Cell Line Bank (Republic of Korea). Cells were cultured in Dulbecco's modified Eagle medium (DMEM; HyClone, USA) supplemented with 10% fetal bovine serum (FBS; HyClone) and 1% penicillin-streptomycin solution (HyClone). The cells were cultured at 37°C in a 5% CO_2_ atmosphere.

### Cell Viability

To evaluate the effects of *L. plantarum* WB326 and *L. brevis* WB2810 on cell viability, an MTT assay was conducted, as described by Lee *et al*. [13] with some modifications. B16F10 melanoma cells were seeded in a 96-well plate at a concentration of 5 × 10^3^ cells/well and incubated at 37°C for 24 h, followed by treatment with *L. plantarum* WB326 and *L. brevis* WB2810. After a 24 h incubation period, the culture medium was aspirated, and the cells were washed once with PBS. Next, MTT solution (0.5 mg/ml) was added to each well, and the cells were incubated for an additional 2 h. The solution was then removed, and dimethyl sulfoxide (DMSO) was used to solubilize the formazan crystals. Absorbance was measured at 570 nm using a microplate reader.

### Intercellular Tyrosinase Activity

The effects of *L. plantarum* WB326 and *L. brevis* WB2810 on cellular tyrosinase activity in B16F10 melanoma cells were assessed using a previously described method [14]. B16F10 cells were seeded into a 6-well plate at a density of 1 × 10^5^ cells/ml and incubated at 37°C for 24 h. Subsequently, the cells were treated with α-melanocyte stimulating hormone (α-MSH; 200 nM, Sigma-Aldrich, USA) along with the samples (1 mg/ml). After 48 h of incubation, the culture medium was aspirated, and each well was washed twice with PBS. The cells were then lysed using 200 μl of 0.1 M sodium phosphate buffer (pH 6.8) containing 1% Triton X-100. The lysate was centrifuged at 18,000 ×*g* for 30 min, and 40 μl of the supernatant was mixed with 160 μl of 5 mM DOPA (dissolved in 0.1 M sodium phosphate buffer). After incubating at 37°C for 1 h, cellular tyrosinase activity was assessed by measuring the absorbance at 475 nm.

### Melanin Synthesis

The melanin content assay was conducted as described by Park *et al*. [14]. B16F10 melanoma cells were seeded in 6-well plates and incubated at 37°C for 24 h. After treating the cells with α-MSH (200 nM) and samples (1 mg/ml), they were further incubated for 48 h. Subsequently, the cells were detached using trypsin-ethylenediamine tetraacetic acid (EDTA) solution (HyClone), followed by centrifugation at 12,000 ×*g* for 10 min. The cell pellet was dissolved in 1 N NaOH and 10% DMSO in a water bath at 80°C for 1 h. Melanin content was quantified by measuring the absorbance at 405 nm.

### Quantitative Real-Time Polymerase Chain Reaction (qRT-PCR)

qRT-PCR was utilized following the methodology outlined in a previous study, with some modifications [14]. B16F10 melanoma cells were seeded in a 6-well plate at a density of 1 × 10^5^ cells/ml and cultured for 24 h before being treated with α-MSH (200 nM) and LAB (1 mg/ml). After incubation for an additional 48 h, total RNA was isolated. Total RNA was extracted using TRIzol (Invitrogen, Thermo Fisher Scientific Inc., USA), followed by cDNA synthesis using a RevertAid First Strand cDNA Synthesis Kit (Bioline, UK). For qRT-PCR, cDNA was combined with SYBR Green PCR Master Mix and specific primers. The RT-PCR protocol involved an initial polymerase activation step at 95°C for 2 min, followed by 40 cycles of denaturation at 95°C for 20 s, annealing at 65°C for 20 s, and extension at 72°C for 30 s. The cycle threshold (Ct) value was normalized to that of the housekeeping gene β-actin, and the relative gene expression level was calculated using the 2^-ΔΔCt^ method. The PCR primer sequences are listed in Table 1.

### DOPA Staining

DOPA staining was performed to observe the effects of *L. plantarum* WB326 and *L. brevis* WB2810 on tyrosinase activity and melanin synthesis in B16F10 cells [15]. B16F10 cells were seeded at a density of 1 × 10^5^ cells/ml in a 6-well plate and incubated at 37°C for 24 h. The cells were treated with α-MSH (200 nM) and samples (1 mg/ml), followed by a 48 h incubation period. The cells were washed with PBS, fixed in formalin solution for 20 min, and rinsed three times with PBS. The cells were then incubated with 5 mM DOPA at 37°C in the dark for 3 h. The fixed cells were examined using a DS-Ri2 digital camera (Nikon Co., Ltd., Japan).

### Western Blot

B16F10 cells were cultured in a 6-well plate with a density of 1 × 10^5^ cells/ml overnight, followed by treatment with α-MSH (200 nM) and samples (1 mg/ml). Protein extraction from B16F10 cells was performed using a lysis buffer containing protease/phosphatase inhibitors from iNtRON Biotechnology (Republic of Korea). Fifteen micrograms of each protein sample were separated using 10% sodium dodecyl sulfate-polyacrylamide gel electrophoresis (SDS-PAGE) and subsequently transferred to a polyvinylidene fluoride (PVDF) membrane. The membranes were blocked using 5% skim milk in Tris-buffered saline with 1% Tween 20 (TBST) for 1 h and then incubated with specific primary antibodies (CREB, p-CREB, MITF, p-p38, p-JNK, p-ERK, p-p65, p-c-Jun, p38, JNK, and ERK) from Cell Signaling Technology Inc. (USA) at 4°C for 16–24 h. After another rinse with TBST, protein bands were detected using an enhanced chemiluminescence solution, and images were captured by exposing the PVDF membranes to an X-ray film. GAPDH was used as the loading control for normalization [16].

### High-Performance Liquid Chromatography (HPLC)

The organic acids produced by the LAB strains were analyzed using high-performance liquid chromatography (HPLC). Each LAB strain was grown in MRS broth for 24 h. Following centrifugation (12,000 ×*g*, 10 min), the supernatants from each culture were filtered through a 0.2 μm membrane filter. The Waters 2487 HPLC system equipped with an Agilent Polaris C18 column (4.6 mm × 250 mm, 5 μm), auto-sampler, and UV detector was utilized to analyze the supernatant, including lactic acid, acetic acid, and propionic acid. Isocratic elution was conducted using 25 mM potassium phosphate buffer (pH 2.5) at a flow rate of 1 ml/min. The chromatograms were recorded at 210 nm [17]. The concentrations of organic acids were determined by constructing a calibration curve using an external standard method with five different concentrations of the standard mixtures.

### Antioxidant Activity of Postbiotics of LAB Strains

DPPH (2,2-diphenyl-1-picrylhydrazyl) radical scavenging activity was measured as described by Bock *et al*.[18] with some modifications. 200 μl of LAB (1 mg/ml) were mixed with 200 μl of DPPH solution (0.1 mM) dissolved in ethyl alcohol. After incubation at room temperature for 30 min, the mixture was centrifuged at 14,240 ×*g* for 1 min. The absorbance of the supernatant was measured at 517 nm. The radical scavenging activity was determined using Eq. (1).

Radical scavenging activity (%) = (1-A_sample_/A_control_)×100 (1)

The ABTS (2,2'-azino-bis-(3-ethylbenzothiazoline-6-sulfonic acid) diammonium salt) radical scavenging activity was assessed following the method described by Bock *et al*. [18] with modifications. Initially, a solution containing 14 mM ABTS and 5 mM potassium persulfate was prepared in distilled water at a 1:1 ratio and incubated at 25°C for 16–24 h in the dark. The resulting ABTS solution was diluted with distilled water to achieve a final absorbance of 0.7 ± 0.05 at 734 nm. Two hundred microliters of LAB (1 mg/ml) were mixed with 800 μl of the diluted ABTS solution and incubated at room temperature for 15 min. After centrifugation at 14,240 ×*g* for 1 min, the absorbance of the supernatant was measured at 734 nm. The radical scavenging activity was determined using Eq. (1).

### Statistical Analysis

All experimental results were obtained from triplicate measurements and are presented as mean ± standard deviation. Statistical analysis was performed using one-way analysis of variance (ANOVA) with Duncan’s multiple range test, and Student’s *t*-test was used to verify significant differences. Statistical significance was defined as *p* < 0.05. Data analysis was conducted using SPSS software (IBM, USA).

## Results and Discussion

### Effects of Postbiotics of *L. plantarum* WB326 and *L. brevis* WB2810 on Cell Viability

**Fig. 1:**
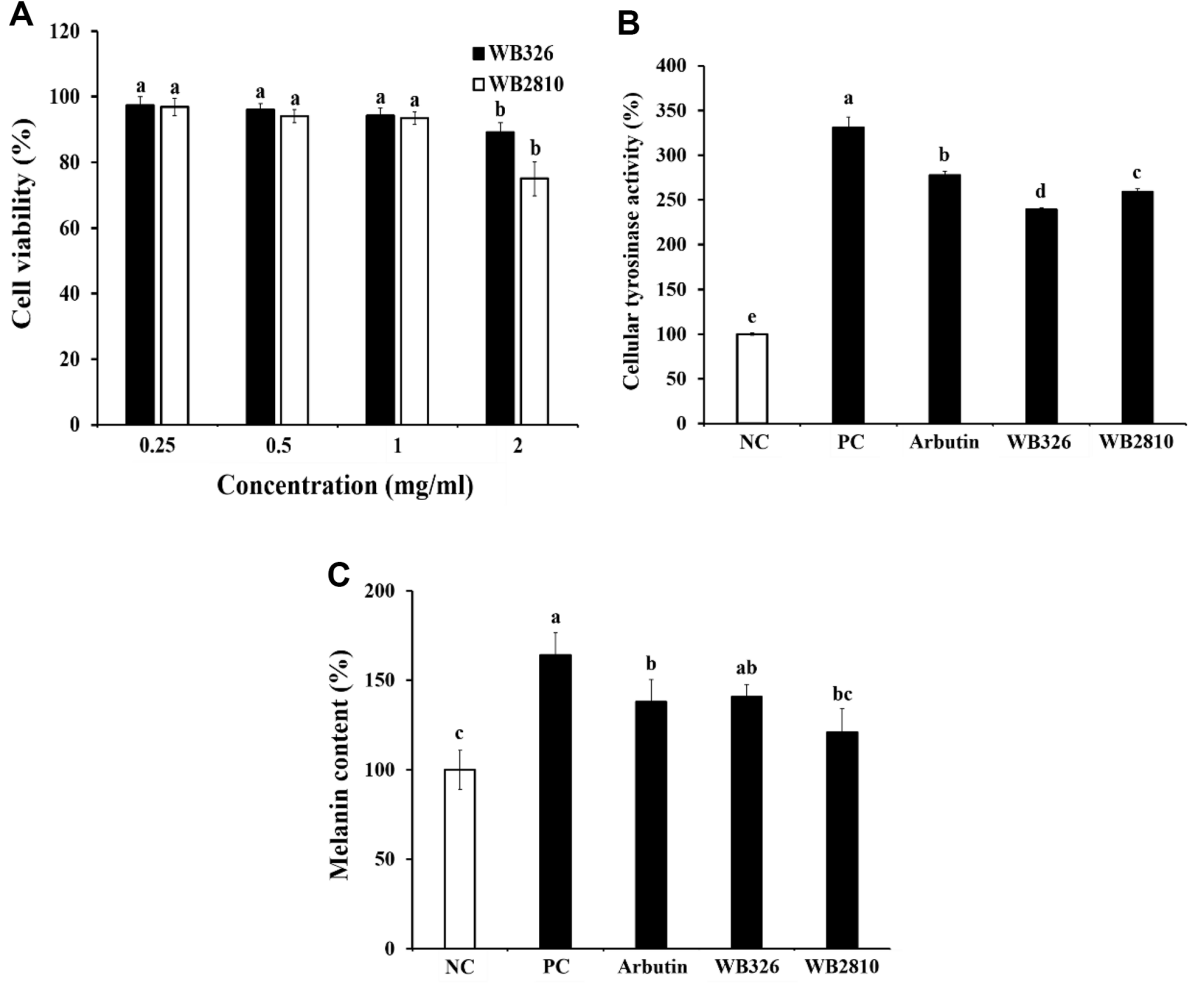
Effects of postbiotic LAB strains on cell viability, tyrosinase activity, and melanin production in α-MSH-induced B16F10 cells. (**A**) Cell viability, (**B**) cellular tyrosinase activity, (**C**) melanin content. NC, negative control without α-MSH; PC, positive control with α-MSH; Arbutin, arbutin with α-MSH; WB326, *L. plantarum* WB326 with α-MSH; WB2810, *L. brevis* WB2810 with α-MSH. Arbutin and each sample were treated 1 mM and 1 mg/ml, respectively. Data are presented as mean ± standard deviation of triplicate experiments. Different letters on error bars represent significant differences (*p* < 0.05).

The lactic acid bacteria isolated from kimchi were utilized in this study due to their characteristics of acid resistance, bile tolerance, and intestinal adhesion ability (data not shown). The screening of strains effective in antimelanogenic effect was conducted with 10 strains, and finally, *L. plantarum* WB326 and *L. brevis* WB2810 used in the experiment were found to be effective in antimelanogenic. B16F10 melanoma cells were utilized to study how *L. plantarum* WB326 and *L. brevis* WB2810 affect cell viability, with the goal of determining whether there is a connection between their anti-melanogenic effects and cytotoxicity. *L. plantarum* WB326 and *L. brevis* WB2810 showed no toxicity at a concentration of 1 mg/ml (Fig. 1A). The following experiments used concentrations of *L. plantarum* WB326 and *L. brevis* WB2810 that showed no cytotoxic effects, thereby ensuring that their anti-melanogenic properties were not compromised.

### Effects of Postbiotics of *L. plantarum* WB326 and *L. brevis* WB2810 on Intercellular Tyrosinase Activity

α-MSH is a critical intrinsic factor in melanogenesis and is regulated by pigmentation enzymes TYR, TRP-1, and TRP-2 [19, 20]. To assess the anti-melanogenic potential of *L. plantarum* WB326 and *L. brevis* WB2810, the impact on tyrosinase production was examined in α-MSH induced B16F10 melanoma cells. In the α-MSH-treated group, tyrosinase activity was significantly increased compared to that in the untreated group. However, treatment with *L. plantarum* WB326 or *L. brevis* WB2810 significantly inhibited tyrosinase activity. *L. plantarum* WB326 and *L. brevis* WB2810 exhibited more potent effects than arbutin (Fig. 1B).

Tyrosinase facilitates the oxidation of L-tyrosine to 3,4-dihydroxyphenyl-L-alanine (L-DOPA), which is the initial and rate-limiting step in melanogenesis [21]. L-Tyrosine or L-DOPA undergoes hydroxylation, followed by the conversion of L-DOPA into DOPA quinone. The inhibition of tyrosinase activity is essential for reducing the production of L-DOPA from L-tyrosine, which is a critical step in preventing melanin formation. Therefore, assessing tyrosinase inhibitory effects is important when evaluating skin-whitening products [22, 23].

### Effects of Postbiotics of *L. plantarum* WB326 and *L. brevis* WB2810 on Melanin Synthesis

Skin pigmentation occurs primarily due to melanocytes in the basal layer of the skin, which are activated by UV radiation. When stimulated, keratinocytes release α-MSH, which is a small peptide hormone [23]. Subsequently, when exposed to UV radiation, keratinocytes induce the secretion of α-MSH to boost melanin production in epidermal melanocytes [21]. Melanin shields skin cells from harm caused by external stimuli such as UV rays and helps eliminate toxic substances [24].

*L. plantarum* WB326 and *L. brevis* WB2810 were tested on melanin production in α-MSH-induced B16F10 melanoma cells for their anti-melanogenic potential. Melanin production in the α-MSH treated group showed a significant increase compared to the untreated group; however, this increase was notably suppressed by *L. plantarum* WB326 and *L. brevis* WB2810. Furthermore, *L. plantarum* WB326 and *L. brevis* WB2810 exhibited similar or superior effects to those of arbutin (Fig. 1C).

### Effects of Postbiotics of *L. plantarum* WB326 and *L. brevis* WB2810 on mRNA Expression of Melanogenic Enzyme

**Fig. 2:**
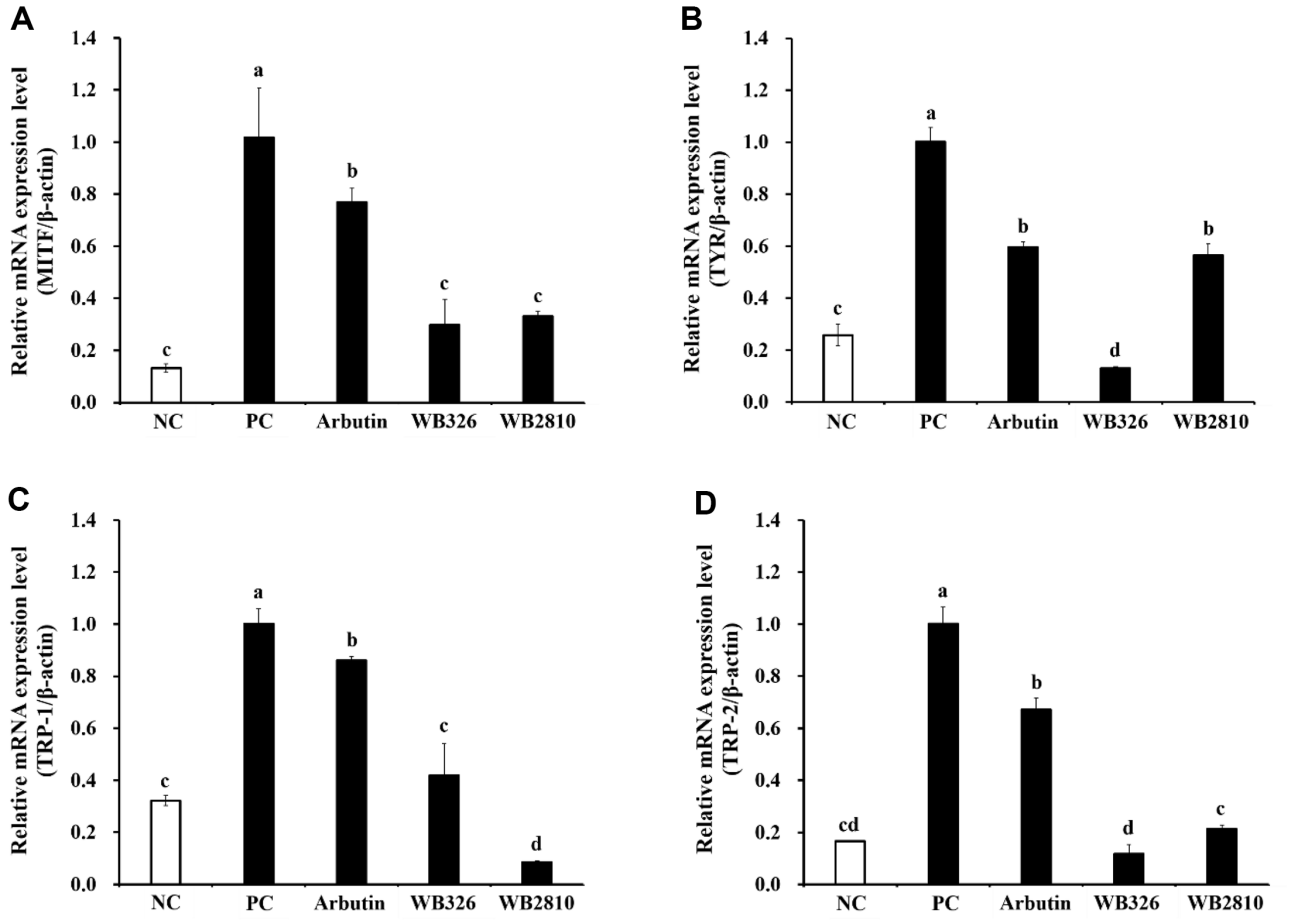
Effects of postbiotic LAB strains on mRNA expression of melanogenic enzyme in α-MSH stimulated B16F10 cells. (**A**) MITF (microphthalmia-associated transcription factor), (**B**) TYR (tyrosinase), (**C**) TRP-1 (tyrosinaserelated protein-1), (**D**) TRP-2 (tyrosinase-related protein-2). NC, negative control without α-MSH; PC, positive control with α- MSH; Arbutin, arbutin with α-MSH; WB326, *L. plantarum* WB326 with α-MSH; WB2810, *L. brevis* WB2810 with α-MSH. Arbutin and each sample were treated at 1 mM and 1 mg/ml, respectively. Data are presented as mean ± standard deviation of triplicate experiments. Different letters on error bars represent significant differences (*p* < 0.05).

An experiment was conducted to determine whether *L. plantarum* WB326 and *L. brevis* WB2810 affect the expression of melanogenic enzyme genes. Compared to the group that did not receive any treatment (control), the group treated with α-MSH showed a significant increase in the levels of MITF, TYP, TRP-1, and TRP-2. In contrast, the groups treated with *L. plantarum* WB326 and *L. brevis* WB2810 showed the lowest levels of melanogenic enzymes, including MITF, TYP, TRP-1, and TRP-2 (Fig. 2).

The activities of tyrosinase, TRP-1, and TRP-2 are controlled by MITF [23]. MITF plays a crucial role in melanin production and distribution by regulating important melanogenic proteins, such as TYR, TRP-1, and TRP-2 [22]. As a transcription factor, MITF binds to a highly conserved gene sequence known as the M-box, which is found in the promoter region of TYR, TRP-1, and TRP-2 [25]. TRP-2 functions as a dopachrome tautomerase, facilitating the conversion of dopachrome to 5,6-dihydroxyindole-2-carboxylic acid (DHICA), while TRP-1 oxidizes DHICA to generate carboxylate indolequinone [24]. This interaction influences the protein expression of TYR, TRP-1, and TRP-2 by increasing the transcription of genes related to the TYR family [25].

### Effect of Melanoma Cells Treated With Postbiotics of *L. plantarum* WB326 and *L. brevis* WB2810 Using DOPA Staining

Melanocytes are specialized cells primarily located in the basal layer of the skin, where they produce melanin within organelles called melanosomes. Melanosomes serve as sites for melanogenesis, the biochemical process responsible for the synthesis of melanin pigments [26, 27]. The produced melanin is then transferred from melanocytes to specific epidermal cells called keratinocytes via dendrites [28]. Within these keratinocytes, melanin forms a protective cap-like structure around the nucleus, scavenging free radicals and protecting the cellular proteins [28].

**Fig. 3:**
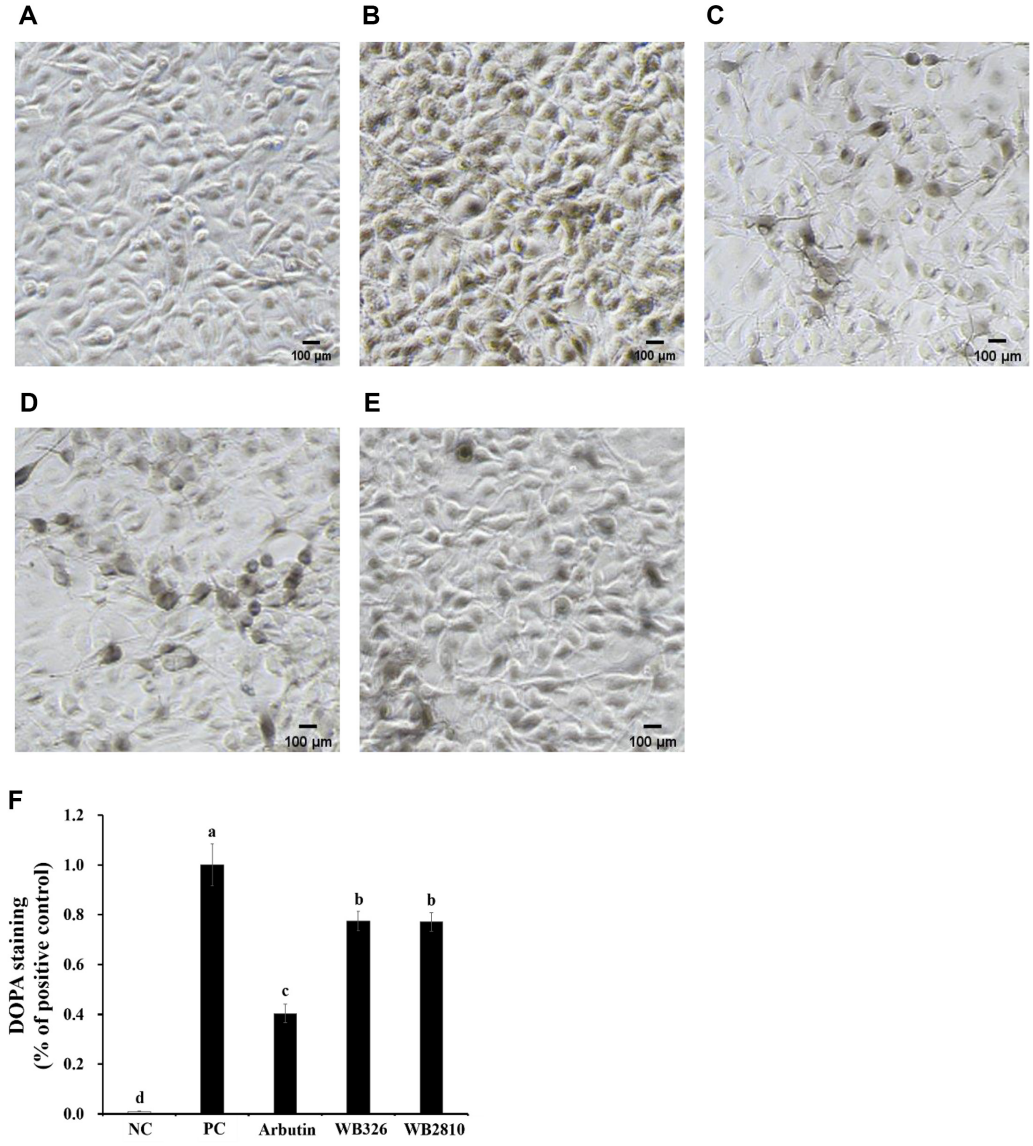
Observation of α-MSH stimulated B16F10 cells treated with postbiotic LAB strains. (**A**) Control, (**B**) control with α-MSH, (**C**) arbutin with α-MSH, (**D**) *L. plantarum* WB326 with α-MSH, (**E**) *L. brevis* WB2810 with α-MSH, (**F**) relative results of DOPA staining using imageJ. Arbutin and each sample were treated at 1 mM and 1 mg/ml, respectively. The black scale bar means 100 μm at ×200 magnification. Data are presented as mean ± standard deviation of triplicate experiments. Different letters on error bars represent significant differences (*p* < 0.05).

As shown in Fig. 3, the effects of *L. plantarum* WB326 and *L. brevis* WB2810 on ROS generation in B16F10 melanoma cells stimulated by α-MSH were evaluated. Under the microscope, when treated with α-MSH, melanin synthesis and dendrite formation were observed. However, treatment with *L. plantarum* WB326 and *L. brevis* WB2810 led to a significant reduction in melanin formation, and these strains showed an inhibitory effect similar to that of arbutin. These results support earlier findings from tyrosinase activity and melanin synthesis experiments.

### Effects of Postbiotics of *L. plantarum* WB326 and *L. brevis* WB2810 on CREB/MITF Signaling Pathway

**Fig. 4:**
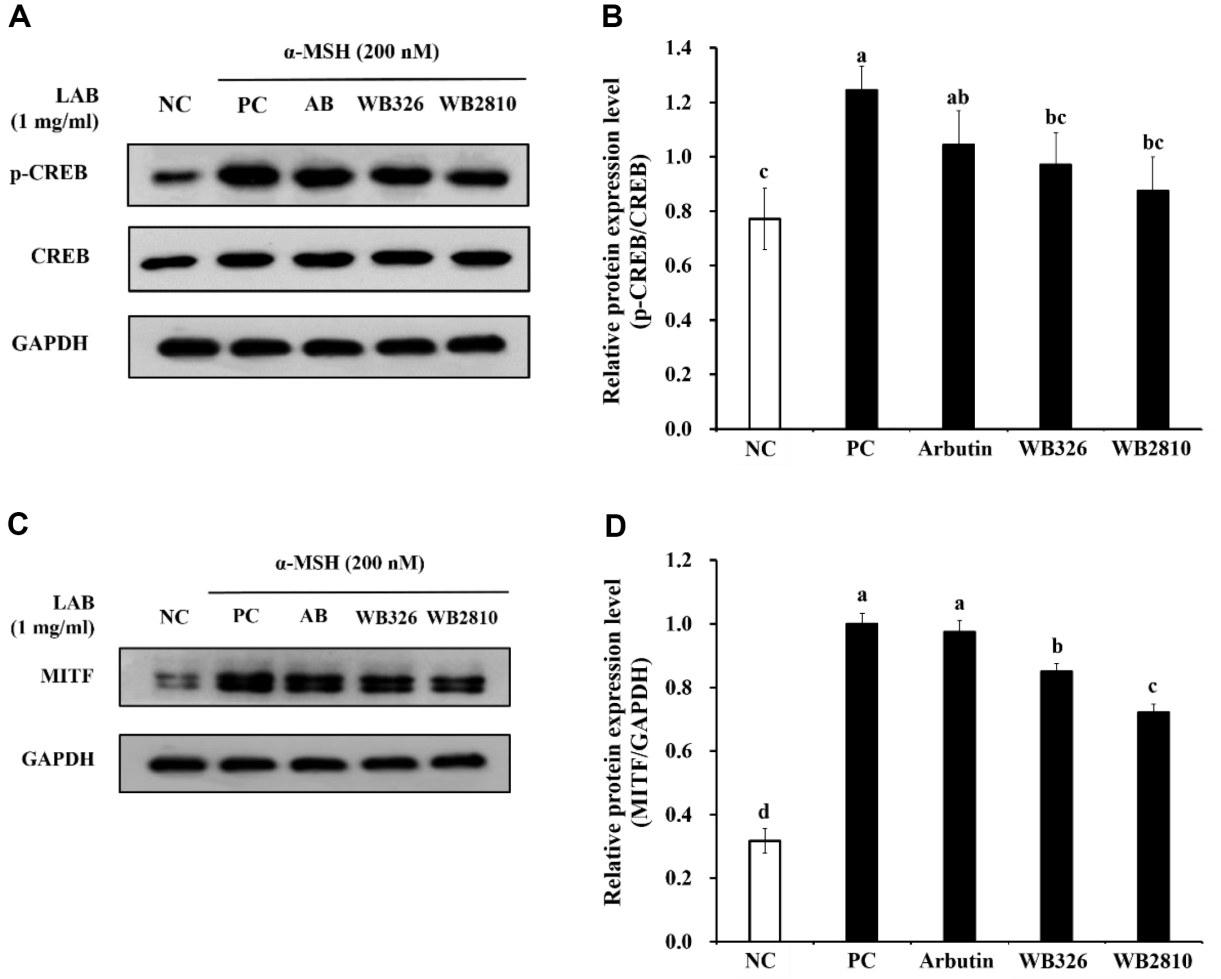
Anti-melanogenic effects of postbiotic LAB strains on the CREB/MITF protein expression in B16F10 cells. (**A**) Image of CREB using western blotting assay, (**B**) Relative p-CREB/CREB, (**C**) Image of MITF using western blotting assay, (**D**) Relative MITF/GAPDH. NC, negative control without α-MSH; PC, positive control with α-MSH; AB, arbutin with α- MSH; WB326, *L. plantarum* WB326 with α-MSH; WB2810, *L. brevis* WB2810 with α-MSH. Arbutin and each sample were treated at 1 mM and 1 mg/ml, respectively. Data are presented as mean ± standard deviation of triplicate experiments. Different letters on error bars represent significant differences (*p* < 0.05).

In order to determine whether proinflammatory factors were downregulated with CREB/MITF signaling, the impact of *L. plantarum* WB326 and *L. brevis* WB2810 was examined in α-MSH induced B16F10 melanoma cells. Upon α-MSH stimulation, significant phosphorylation of CREB and MITF was observed. However, *L. plantarum* WB326 and *L. brevis* WB2810 decreased CREB phosphorylation and MITF compared to the positive control treated with α-MSH (Fig. 4). Therefore, *L. plantarum* WB326 and *L. brevis* WB2810 modulate the CREB/MITF signaling pathway to suppress melanogenesis.

In melanosomes, CREB phosphorylation is controlled by the cAMP/protein kinase A (PKA) cascade, which is crucial for melanin synthesis [29]. MITF expression is mainly regulated by cAMP/PKA-dependent activation, which results in the expression of melanogenic enzymes that ultimately promote melanin production [8]. MITF plays a critical role in regulating the expression of melanogenic proteins and is influenced by signals that enhance its expression, including those mediated by MC1R activation and α-MSH binding to specific MC1R. MC1R stimulation leads to elevated cAMP levels, which subsequently stimulate MITF expression [9].

### Effects of Postbiotics of *L. plantarum* WB326 and *L. brevis* WB2810 on MAPK Signaling Pathway

**Fig. 5:**
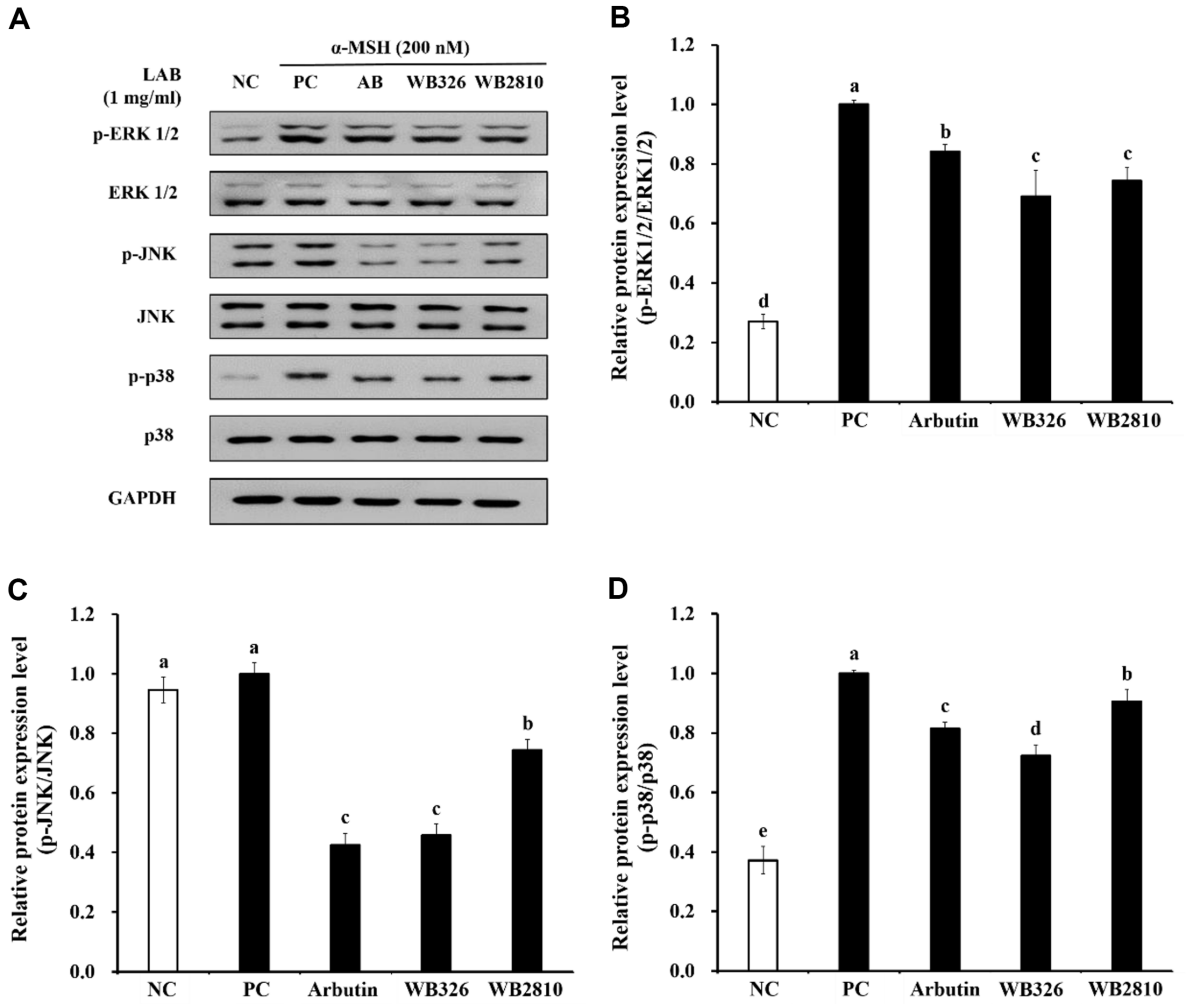
Anti-melanogenic effects of postbiotic LAB strains on the MAPK pathway protein expression in B16F10 cells. (**A**) Image of MAPK using western blotting assay, Relative expression of (**B**) p-ERK1/2/ERK1/2, (**C**) p-JNK/ JNK, (**D**) p-p38/p38. NC, negative control without α-MSH; PC, positive control with α-MSH; AB, arbutin with α-MSH; WB326, *L. plantarum* WB326 with α-MSH; WB2810, *L. brevis* WB2810 with α-MSH. Arbutin and each sample were treated at 1 mM and 1 mg/ml, respectively. Data are presented as mean ± standard deviation of triplicate experiments. Different letters on error bars represent significant differences (*p* < 0.05).

A significant amount of phosphorylation of MAPKs was observed upon α-MSH stimulation (Fig. 5). The reduction in ERK1/2, p38, and JNK phosphorylation correlated with the anti-melanogenic effects of *L. plantarum* WB326 and *L. brevis* WB2810. These findings suggest that *L. plantarum* WB326 and *L. brevis* WB2810 inhibit MAPK activation and exert their anti-melanogenic properties.

ERK or JNK activation through phosphorylation initiates MITF expression, leading to its degradation and the consequent reduction of melanogenesis. Conversely, ERK activation can phosphorylate CREB, enabling it to bind to the CRE consensus motif in the MITF promoter region and enhance MITF expression. Additionally, p38 phosphorylation triggers MITF expression, promoting the upregulation of melanogenesis-related proteins and ultimately stimulating melanin synthesis [31].

### Quantification of Organic Acid Using HPLC Analysis

**Fig. 6:**
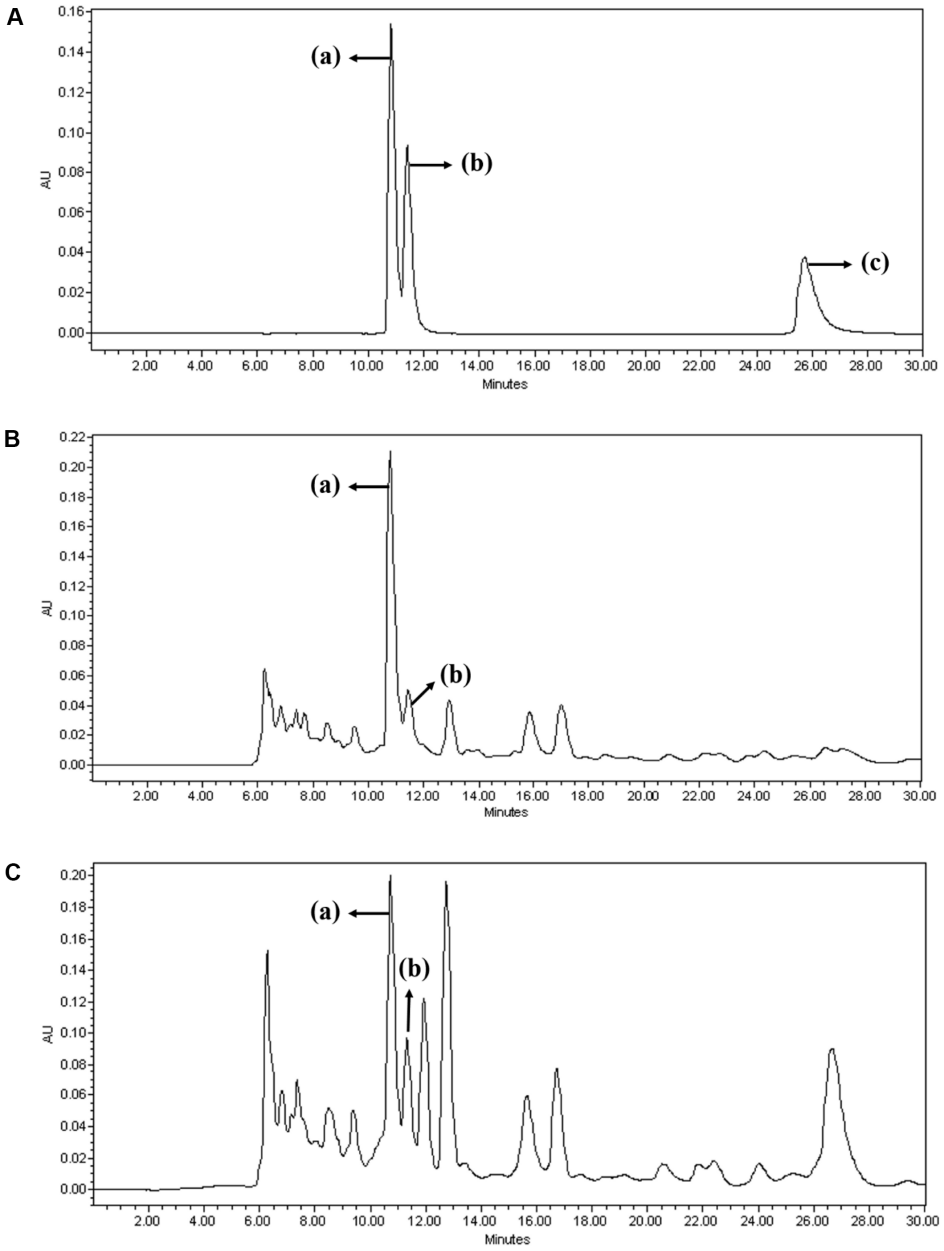
Analysis of organic acids of postbiotic LAB strains with HPLC. (**A**) Standards of organic acid, (**B**) Supernatants of *L. plantarum* WB326, (**C**) Supernatants of *L. brevis* WB2810. (a) Lactic acid; (b) acetic acid; (c) propionic acid.

Fig. 6 shows the results of HPLC analysis of the organic acid standards and LAB samples. Lactic, acetic, and propionic acids were used as standards. In the sample supernatant analysis, neither *L. plantarum* WB326 nor *L. brevis* WB2810 showed the presence of propionic acid. In contrast, lactic and acetic acids were detected, with lactic acid being the most abundant (Table 2). Compared to the amounts of organic acids reported in Hu *et al*. [35] and Seo *et al*. [36], higher levels of organic acids were found in *L. plantarum* WB326 and *L. brevis* WB2810.

LAB and lactic acid are used to promote skin health and explore the potential use of LAB-derived metabolites/components for skin-whitening applications [4]. In cosmetics, lactic acid exerts a skin-brightening effect by inhibiting tyrosinase production, and its ability to retain moisture contributes to its moisturizing effect [4]. Lactic acid is included in the natural moisturizing factor (NMF), which assists in retaining moisture within the skin and significantly contributes to the physical characteristics of the stratum corneum [32]. Halstead *et al*. [33] reported that acetic acid exhibited impressive bactericidal effects when tested *in vitro*. These effects result from the capacity of acetic acid to decrease the pH levels, which creates an environment unfavorable for pathogen growth [32].

### Antioxidant Activity of Postbiotics of *L. plantarum* WB326 and *L. brevis* WB2810

Free radicals and reactive oxygen species play crucial roles in accelerating skin aging by inducing oxidative stress in skin cells, leading to increased melanin production and wrinkles [23]. Antioxidants are employed in the cosmetics industry to prevent or delay skin aging by inhibiting oxidation through the removal of free radicals, reactive oxygen species (ROS), and oxidation-catalytic metals [4].

**Fig. 7:**
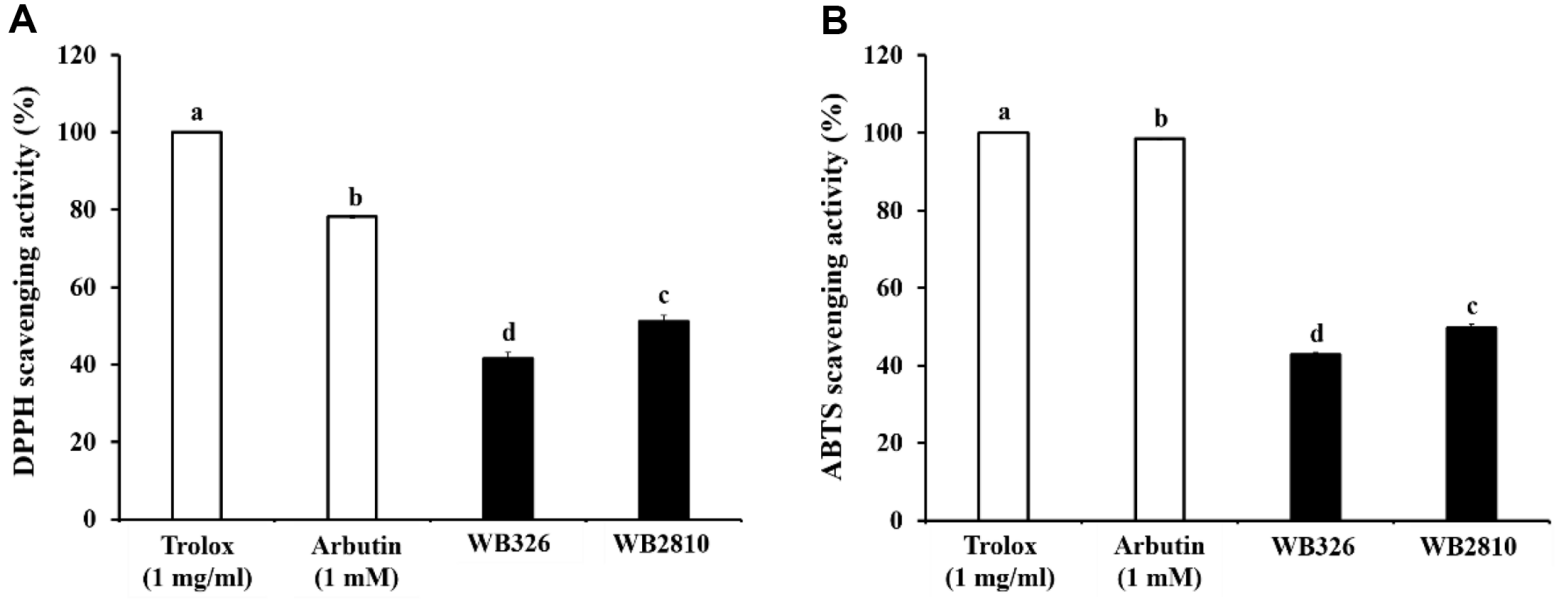
Antioxidant activities of postbiotic LAB strains. (**A**) DPPH radical scavenging activity, (**B**) ABTS radical scavenging activity. WB326, *L. plantarum* WB326; WB2810, *L. brevis* WB2810. Arbutin and each sample were treated 1 mM and 1 mg/ml, respectively. Data are presented as mean ± standard deviation of triplicate experiments. Different letters on error bars represent significant differences (*p* < 0.05).

Antioxidant activities of the LAB strains were assessed using DPPH and ABTS assays (Fig. 7). In the DPPH assay, the DPPH scavenging activities of *L. plantarum* WB326 and *L. brevis* WB2810 were 41.60% and 51.10%, respectively. The Trolox and arbutin levels were 100% and 79%, respectively. In the ABTS assay, *L. plantarum* WB326 (42.84%) and *L. brevis* WB2810 (49.76%) showed radical scavenging activity. The Trolox and arbutin levels were 100% and 98.44%, respectively.

This study revealed that *L. plantarum* WB326 and *L. brevis* WB2810 have whitening effects. *L. plantarum* WB326 and *L. brevis* WB2810 effectively inhibited tyrosinase and melanin synthesis. In addition, *L. plantarum* WB326 and *L. brevis* WB2810 effectively inhibited melanogenic enzyme expression and protein activity via the CREB/MITF and MAPK pathways, which are associated with whitening and pigmentation inhibition, respectively. *L. plantarum* WB326 and *L. brevis* WB2810 were analyzed for their organic acid content using HPLC, and their antioxidant capacities were assessed to investigate their effectiveness in reducing ROS. In summary, the postbiotic *L. plantarum* WB326 and *L. brevis* WB2810 exhibited potential as whitening-effective strains and could be considered functional ingredients in the cosmetics industry.

## Figures and Tables

**Table 1:** Primer sequences used in RT-PCR.

Primer^a^		Primer sequence (5’-3’)
MITF	Forward	TTACCAACAACCTCGGCACCAT
	Reverse	CTCCTGGCGACACTGATGACA
TYR	Forward	CCTCCTGGCAGATCATTTGT
	Reverse	GGTTTTGGCTTTGTCATGGT
TRP-1	Forward	TTGCTGTAGTGGCTGCGTTGTT
	Reverse	AGGAGAGGCTGGTTGGCTTCAT
TRP-2	Forward	GCAAGAGATACACGGAGGAAG
	Reverse	CTAAGGCATCATCATCATCACTAC
β-Actin	Forward	GTGGGCCGCCCTAGGCACCAG
	Reverse	GGAGGAAGAGGATGCGGCAGT

^a^MITF, microphthalmia-associated transcription factor (MITF); TYR, tyrosinase; TRP-1, tyrosinase-related protein-1; TRP-2, tyrosinase-related protein-2. *β-Actin* was utilized as the housekeeping gene.

**Table 2:** Organic acid production by postbiotic LAB strains.

Lactic acid bacteria	Lactic acid (mg/l)	Acetic acid (mg/l)
*L. plantarum* WB326	26,980.07 ± 1,530.52*	2,861.46 ± 499.28
*L. brevis* WB2810	13,214.66 ± 856.56*	3,276.81 ± 230.05

Data are indicated as mean ± standard deviation of triplicate experiments. For different strains in the same experiment, the means with superscript letters (*) were significant (*p* < 0.05, Student’s *t*-test).
